# The role of dendritic cells regulated by HMGB1/TLR4 signalling pathway in myocardial ischaemia reperfusion injury

**DOI:** 10.1111/jcmm.14192

**Published:** 2019-02-19

**Authors:** Jiyang Xue, Hanwei Ge, Zhiyong Lin, Hanlei Wang, Wei Lin, Yong Liu, Guowei Wu, Jie Xia, Qifeng Zhao

**Affiliations:** ^1^ Department of Cardiovascular and Thoracic Surgery Children's Heart Center, The Second Affiliated Hospital & Yuying Children's Hospital, Institute of Cardiovascular Development and Translational Medicine, Wenzhou Medical University Wenzhou P.R. China

**Keywords:** dendritic cell, HMGB1, ischaemia/reperfusion injury, signal pathway, TLR4

## Abstract

Inflammatory response plays an important role in ischaemia reperfusion injury (IRI) through a variety of inflammatory cells. Apart from neutrophils, macrophages and lymphocytes, the role of dendritic cells (DCs) in IRI has been noticed. The study was aimed at investigating whether the high‐mobility group protein box‐1/toll like receptor 4 (HMGB1/TLR4) signalling pathway regulate the migration, adhesion and aggregation of DCs to the myocardium, induce DCs activation and maturation, stimulate the expression of surface costimulatory molecules and participate in myocardial IRI. In vivo, migration, adhesion, and aggregation of DCs was enhanced; the expression of peripheral blood DCs CD80 and CD86, myocardial adhesion molecules were increased; and the infarct size was increased during myocardial ischaemia reperfusion injury myocardial ischemic/reperfusion injury (MI/RI). These responses induced by MI/RI were significantly inhibited by HMGB1 specific neutralizing antibody treatment. Cellular experiments confirmed that HMGB1 promoted the release of inflammatory cytokines through TLR4/MyD88/NF‐κB, upregulated CD80 and CD86 expression, mediated the damage of cardiomyocytes and accelerated the apoptosis. Our results indicate that DCs activation and maturation, stimulate the expression of surface costimulatory molecules by promoting the release of inflammatory factors through NF‐κB pathway and participate in myocardial IRI.

## 1 INTRODUCTION

Over the past decades, with the development of thrombolytic therapy, cardiovascular surgery under cardiopulmonary bypass and percutaneous transluminal coronary angioplasty, the survival rate of heart disease patients has been significantly improved. However, myocardial ischaemia reperfusion injury myocardial ischemic/reperfusion injury (MI/RI) remains a major obstacle to the treatment of cardiovascular disease.^1^, ^2^, ^3^ Reducing MI/RI has been a hot research topic in cardiovascular field. The pathogenesis of MI/RI is complex, among which local and systemic inflammatory response is the most prominent characteristic.^4^ The pathogenesis involves the infiltration of inflammatory cells and the production of inflammatory cytokines and chemokines, which leads to substantial damage of myocardium. Apart from neutrophils, macrophages and lymphocytes, the role of dendritic cells (DCs) in ischaemia reperfusion injury (IRI) has been recently received considerable attention by researchers.^5^, ^6^, ^7^

Dendritic cells is a critical component of innate immune and adaptive immune response. It is one of the key inflammatory cells that affect the development and progression of IRI, however, the mechanism involved is perplexing. Literature has emerged that offers contradictory findings, and there is no general agreement on the role of DCs in IRI to date. This is due to its role in regulating immune function, which is closely related to the maturation, activation degree, quantity and local microenvironment of DCs.^8^, ^9^, ^10^ In addition, little is known about its function, occurrence, and interaction between groups. A considerable amount of literature has been published about liver and kidney IRI, however, no previous study has investigated that which signalling pathway mediates DCs in the MI/RI process, so the role of DCs in the pathogenesis of MI/RI needs more researches to explore.^11^ High‐mobility group protein box‐1 (HMGB1) is a specific ligand of toll like receptor 4 (TLR4) and its release into extracellular space or serum after cell necrosis or injury produces a wide range of cellular biological effects,^12^, ^13^, ^14^ while TLR4 is mainly expressed on macrophages, DCs and other cells.^15^ In recent years, studies have confirmed that HMGB1/TLR4 signalling pathway plays an important role in IRI.^16^, ^17^, ^18^ However, it's still not clearly defined whether or not HMGB1 controls the function of DCs through the TLR4 on the DCs, thus affecting the role of DCs in MI/RI. This study is based on rat model of MI/RI, DCs intervention experiment, DCs and myocardial cell co‐culture experiment, HMGB1, HMGB1 antibody and TLR4 antagonists treatment, to explore the effect of HMGB1/TLR4 pathway on DCs function and its mechanism, to provide ideas and theoretical basis for the prevention and treatment of MI/RI.

## 2 MATERIALS AND METHODS

### 2.1 Animals

Male Sprague‐Dawley (SD) rats (6‐8 weeks old, weighing 200‐250 g) provided by the experimental animal center of Wenzhou Medical University (animal license No.: SYXK Zhejiang 2015‐0009) were used to establish an animal model and to isolate and culture of DCs and myocardial cells.

The animal procedures were approved by Wenzhou Medical University Animal Care and Use Committee (No: wydw2014‐0058), which were certified by the Chinese Association of Accreditation of Laboratory Animal Care and were consistent with the Guide for the Care and Use of Laboratory Animals (updated [2011] version of the NIH guidelines). All animals were fed a standard diet and maintained in the controlled environment of the animal center at 25 ± 1°C under a 12 hour light‐dark cycle, and they were allowed free access to food and water.

### 2.2 **Rat model of **MI/RI

Rats were anesthetized by inhaling isoflurane. Peripheral blood was collected by femoral venipuncture. Then the animals were intubated for artificial ventilation with 100% oxygen using a breathing machine (tidal volume 5 mL, frequency 80 per minute) and monitored by Electrocardiogram. Thoracotomy was performed between the sternum and left costa, and then the pericardium was opened. Myocardial ischaemia was induced by ligating the left anterior descending coronary artery (LAD) using a 3‐0 silk suture, and the coronary artery was occluded by pulling on the suture tightly 10 minutes later. After 30 minutes of myocardial ischaemia, reperfusion started by releasing the ligature and removing the tube for 180 minutes. The sham group were anaesthetized and their LAD was threaded but not ligation.

The indications of successful LAD occlusion or reperfusion included ECG and myocardial color.^19^, ^20^, ^21^

### 2.3 Isolation, culture, purification and identification of rat DCs

See the Supplementary Materials and Methods for details.

### 2.4 Isolation, culture, purification and identification of cardiac myocytes in rat

See the Supplementary Materials and Methods for details.

### 2.5 Experimental grouping and processing of in vivo experiments

**Figure 1:**
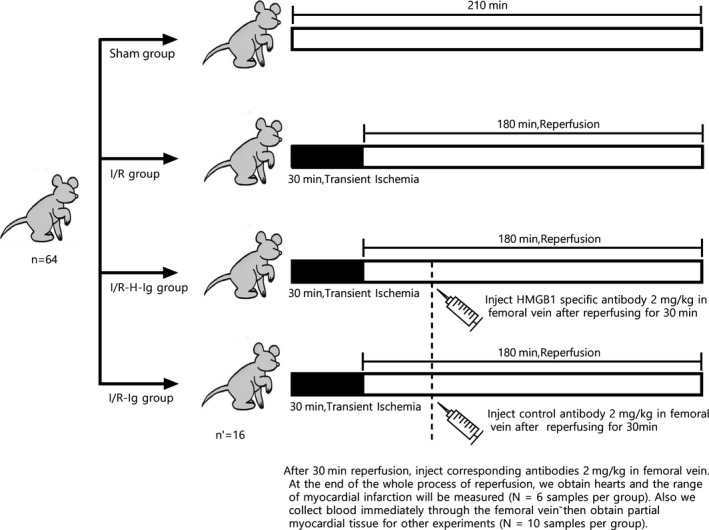
After 30 min reperfusion, inject corresponding antibodies 2 mg/kg in femoral vein. At the end of the whole process of reperfusion, we obtain hearts to measure the range of myocardial infarction (N = 6 samples per group). We also collect blood immediately through the femoral vein，then obtain partial myocardial tissue for other experiments (N = 10 samples per group)

As shown in the Figure 1, SD rats were divided into four groups randomly, with 10 rats in each group. The sham operation group (Sham group): opened the chest, with only a ligation of LAD; the control group (Ischemia‐reperfusion; IR‐C group): subjected to 30 minutes of myocardial ischaemia followed by 180 minutes of reperfusion; HMGB1 specific antibody (ST326052233; SHINO‐TEST Corporation, Japan) treatment group (IR‐H‐Ig group): subjected to 30 minutes of myocardial ischaemia followed by 180 minutes of reperfusion, injected HMGB1 specific antibody 2 mg/kg in femoral vein after reperfusing for 30 minutes; control antibody control group (IR‐Ig group): subjected to 30 minutes of myocardial ischaemia followed by 180 minutes of reperfusion, injected control antibody 2 mg/kg in femoral vein after reperfusing for 30 minutes. The other 24 SD rats were divided into four groups randomly, with six rats in each group. The experimental grouping was the same as above, and the range of myocardial infarction was measured.

### 2.6 Blood collection and tissue harvest

After 180 minutes of reperfusion (group Sham at the corresponding time point), the blood was collected immediately through the femoral vein and used for the detection of inflammatory factors and DCs costimulatory molecules. After collecting the blood, the rat heart was obtained and partial myocardial tissue was fixed with 4% polyoxymethylene, paraffin embedded for pathology and histochemical examination; additional myocardial tissue for protein and gene detection. The myocardial infarct size was assessed by Evan’s Blue and 2,3,5‐triphenyl tetrazolium chloride (TTC) double staining method.

### 2.7 Immunofluorescence

Rat cardiac tissues was conducted using CD1a (orb6188; biorbyt, San Francisco, CA), CD80 (555012; BD, Franklin Lakes, NJ) primary antibody mixture,1:60 diluted Alexa Fluor 594 labeled donkey against rabbit IgG (BYE026; Shanghai Boyun Biotech, Shanghai, China) and 1:60 diluted Alexa Fluor 488 labeling the mixture of donkey against mouse IgG secondary antibody (IgG) (BYE023; Shanghai Boyun Biotech) according to the standard protocol, then observed and photographed under fluorescence microscope after mounting.

### 2.8 Expression of pbDCs costimulatory molecules

After the pbDCs were isolated, cultured and purified, they were incubated with CD11c antibody, then the flow cytometry detection was applied to determine the purity of the DCs. If the purity of cells were higher than 90%, the cells were sampled by flow cytometry and incubated with CD86 (sc‐28347; Santa Cruz), then the CD86 signal intensity was detected by flow cytometry. After incubated with CD80 antibody (sc‐58911; Santa Cruz), then added Fluorescein (FITC) ‐ conjugated Affinipure Goat Anti‐Rat IgG (SA00003‐11; Proteintech, Rosemont, IL) to incubate. Detecting the signal intensity of CD80 by flow cytometry.

### 2.9 Hematoxylin‐eosin staining

Myocardial tissue slices were fixed in 4% paraformaldehyde and subsequently embedded in paraffin. Sections (5 μm thick) were stained with hematoxylin‐eosin (HE) using a standard protocol and analyzed by light microscopy.

### 2.10 Immunohistochemically staining

ICAM‐1 (1:1000 dilution, ab206398; Abcam, Cambridge, UK), P‐selectin (1:1000 dilution, sc‐8419; Santa Cruz), E‐selectin (1:1000 dilution, DF6914; Affinity Biosciences, OH) antibodies were used in paraffin embedded heart sections. The results were analyzed by the multi‐function color cell image analysis system (Image‐Pro Plus V 5.1 software; Media Cybernetics company, Rockville, MD), and system automatically selects 10 meaningful vision, the integral optical density (IOD) was obtained for each view, and 10 views were chosen to calculated the mean IOD.

### 2.11 Myocardial infarct size measurement

The myocardial infarct size was assessed by Evans Blue (E2129; Sigma, St. Louis, MO) and TTC (T8877; Sigma) double staining method. The viable tissue which was stained red and white by TTC was defined as AAR. The non‐ischemic myocardium was stained deep blue by Evans Blue. Infarct area (INF) appeared pale after staining. A percent of infarcted area over total area at risk (INF/AAR ratio, IAR, %) was calculated.^22^

### 2.12 Plasma CK‐MB, cTnI, HMGB1, cytokine (IL‐6, IL‐8, tumor necrosis factor‐α, IL‐12), concentration

For inflammatory markers immunoassay, the serum levels of HMGB1, IL‐6, IL‐8, tumor necrosis factor (TNF)‐α, IL‐12, and CK‐MB, cTnI were measured using a rat Enzyme‐Linked ImmunoSorbent Assay kit (Shanghai Boyun Biotech) in accordance with the manufacturer's instructions.

### 2.13 Western Blot analysis

Myocardial tissue (5 mg) was selected to extract the protein for determination of the protein concentration by bicinchoninic acid. The following primary antibodies were used: 1:300 diluted Rabbit anti HMGB1 (DF3077; Affinity Biosciences), TLR4 (AF7017; Affinity Biosciences) and NF‐κB (AF5006; Affinity Biosciences) primary antibody. In the next day, added 1:3000 diluted secondary antibody, used ECL for coloration, scanned and analyzed the results by the gel imaging system, used the gray ratio of each protein to beta actin to compare and analyze.

### 2.14 RT‐PCR analysis

Total RNA was extracted from myocardial tissue using the TRIzol reagent (15596026; Ambion, Cambridge, UK), and RNA content measured using 260/280 UV spectrophotometry. The same volume of RNA solution was used to reverse transcriptase by the RT‐PCR kit (FSQ‐101; TOYOBO, Kita‐ku, Osaka, Japan). The mRNA expression of HMGB1, TLR4 and NF‐κB were quantified by SYBR Green (170‐8882AP; Bio‐Rad, Hercules, CA) two‐step, real‐time RT‐PCR using CFX96 Touch Real‐Time PCR Detection System. The expression of each gene was normalized to GAPDH mRNA content and calculated using comparative Ct methods. The primers sequence are shown in Table 1.

**Table 1:** Primer sequences for real‐time quantitative PCR

Gene	Forward primer	Reverse primer	Size (bp)
HMGB1	5′‐GCCGGGAGGAGCACAAGAAGAA‐3′	5′‐GCCTTGTCAGCCTTTGCCATATCT‐3′	139
TLR4	5′‐TTATCCAGAGCCGTTGGTGT‐3′	5′‐CCCACTCGAGGTAGGTGTTT‐3′	171
MyD88	5′‐GGCCACCAGGACCACAAGGACAAT‐3′	5′‐TGGGGGCGGAATGTTTTTGTGTG‐3′	142
NF‐κB P65	5′‐CGCGGTTACGGGAGATGTGAAGAT‐3′	5′‐CGGCCAAGTGCAAAGGTGTCTGAT‐3′	212
GAPDH	5′‐ACGGCAAGTTCAACGGCACAGTCA‐3′	5′‐AGCGGAAGGGGCGGAGATGA‐3′	212

HMGB1, high‐mobility group protein box‐1; TLR4, toll like receptor 4.

### 2.15 Doppler echocardiography

Transthoracic echocardiography was performed at the end of the whole process of reperfusion to measure left ventricular internal dimension in systole (LVIDs), left ventricular internal dimension in diastole (LVIDd) and left ventricular ejection fraction (LVEF) in each group by a technician blinded to the groups.^23^

### 2.16 Experimental grouping and processing of the in vitro experiments

mDCs was randomly divided into five groups, each group had three time points of 24, 48 and 72 hours, with three wells in each group. (a) C group: control group, mDCs was added to the complete medium of RPMI l640; (b) H group: HMGB1 (Ab215008; Abcam) was added to the complete medium containing mDCs (Ab215008, Abcam) (1 μg/mL); (c) H‐H‐Ig group: HMGB1 (1ug/mL) was added followed by HMGB1 specific neutralizing antibody (ST326052233; Shino‐Test, Tokyo, Japan) (the final concentration was 1 μg/mL) was added 30 minutes later; (d) H‐TLR4‐A group: HMGB1(1ug/ml) was added followed by TLR4 antagonists (A3850; ApexBio, Boston, MA) (the final concentration was 1 μg/mL) 30 minutes later; (e) H‐Ig group: HMGB1 (1ug/mL) was added followed by control IgG antibody (ST326058471; Shino‐Test) (the final concentration was 1 μg/mL) 30 minutes later.

### 2.17 Test sample collection

After the experiment was completed, culture supernatant was used to detect the cytokine level. RT‐qPCR and Western Blotting were performed to detect the expression of MyD88, NF‐κB gene and protein in DCs; and flow cytometry was performed to detect the expression of costimulatory molecules.

### 2.18 Expression of mDCs costimulatory molecules

The detection method was the same as detecting the expression of pbDCs costimulatory molecules in vivo.

### 2.19 Supernatant IL‐12, TNF‐α, IL‐8, IL‐6 concentration

The detection method was the same as determination of plasma cytokines (IL‐6, IL‐8, TNF‐α, IL‐12) concentration in vivo.

### 2.20 Western Blot analysis

The detection method of MyD88 and NF‐κB protein was the same as in vivo. The primary antibodies of Western Blot Analysis were MyD88 (sc‐74532; Santa Cruz) and NF‐κB (AF5006; Affinity Biosciences).

### 2.21 RT‐qPCR analysis

The detection method of MyD88 and NF‐κB mRNA was the same as in vivo by RT‐qPCR Analysis. The primers sequence of MyD88, NF‐κB are shown in Table 1.

### 2.22 **Co‐culture of mDCs and **cardiac myocytes

The experiment was divided into the normal oxygen and the hypoxia/reoxygenation group, each of which was divided into five subgroups. The corresponding drugs were added according to the requirements of the in vitro experimental grouping and treatment. After that, the culture supernatant was taken for cTnI detection. The cardiac myocytes (CMs) in the lower chamber were collected, and the apoptosis was detected by flow cytometry.^22^ See the Supplementary Materials and methods for details.

### 2.23 Statistical analysis

Data were expressed as mean ± SD. Comparisons between groups were assessed by One‐way ANOVA followed by Bonferroni's post hoc test. All statistical analyses were performed using Graph Pad Prism Software (Version X, La Jolla, CA). The significance level was set at *P < *0.05*.*

## 3 RESULTS

### 3.1 HMGB1‐TLR4 signalling pathway mediates the migration,adhesion and activation of DCs in IR myocardium

**Figure 2:**
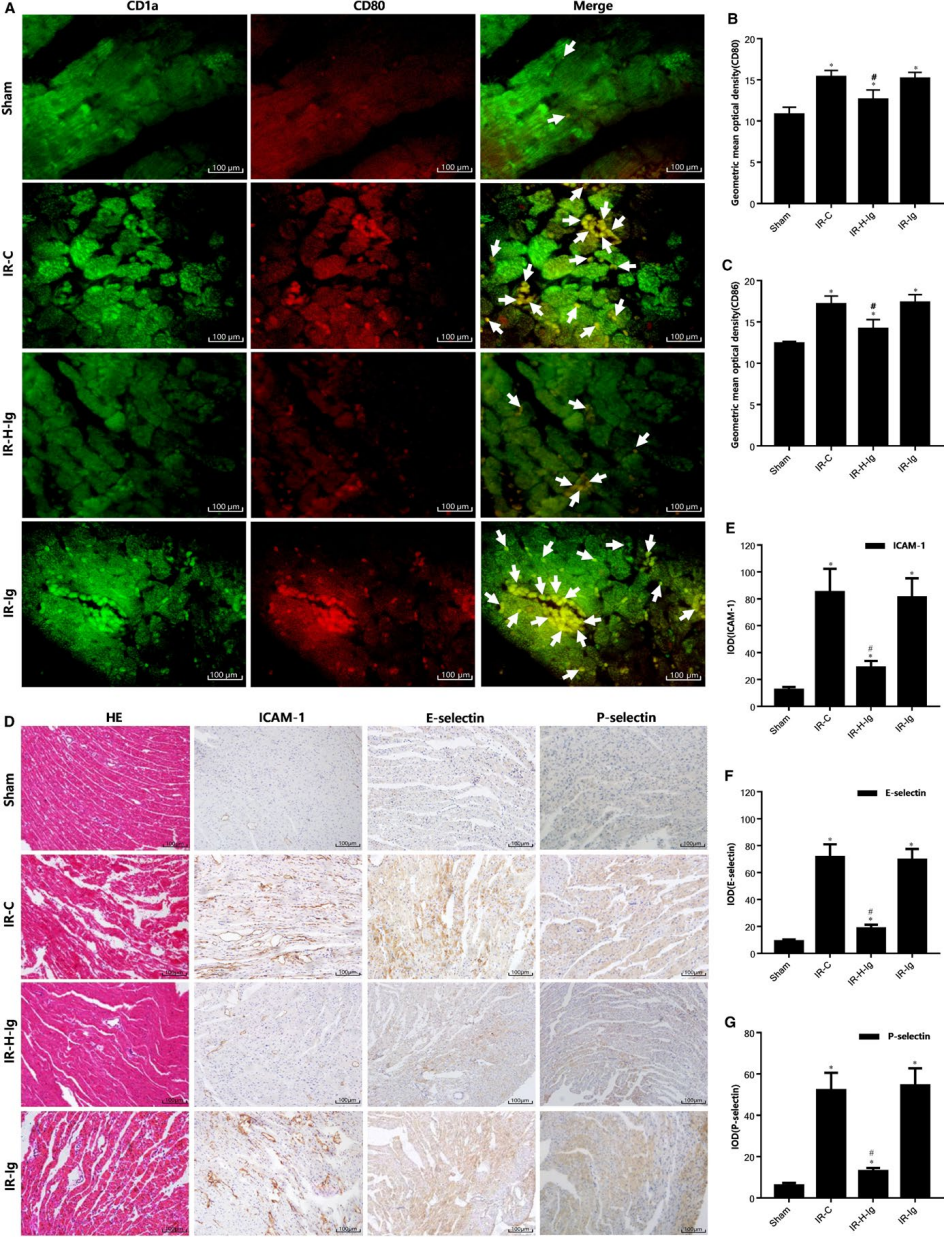
HMGB1‐TLR4 signalling pathway mediates the migration, adhesion and activation of dendritic cells (DCs) in ischaemia‐reperfusion myocardium. A, Double immunofluorescence of DCs in myocardial tissue among all groups (n = 10 for each group). Samples were collected right after IR procedure and staining was performed as described in Section 2. Representative fluorescence images (200×) of the distribution of CD1a (green), of CD80 (red). Arrows indicate co‐localization. (B,C) The geometric mean optical density of CD80, CD86 among all groups (n = 10 for each group) in peripheral blood of rats was detected by flow cytometry. Histological study, immunohistochemical staining in cardiac tissues from rats of different groups (n = 10 for each group). Samples were collected right after IR procedure was over and staining. D, Hematoxylin‐eosin (HE) staining pictures (200×) are shown in the left, immunohistochemical staining pictures (200×) are in the middle and right; (E) integral optical density (IOD) of ICAM‐1; (F) IOD of E‐selectin; (G) IOD of P‐selectin. Scale bars, 100 μm. *P < 0.05, comparisons of IR‐C, IR‐H‐Ig and IR‐Ig groups with Sham group; #*P *< 0.05, comparisons of IR‐H‐Ig and IR‐Ig group with IR‐C group

After MI/RI, there are great number of inflammatory cells in the myocardium, which was confirmed in HE staining pictures (Figure 2D) and early studies.^24^ In rat IR myocardium, Wang^25^ also showed that neutrophil aggregation could be detected after myocardial myeloperoxidase staining. In our rat model, DCs specific marker CD1a and CD80 double fluorescent staining was used to study the increase of aggregation and adhesion of CD1a+CD80+ cells to myocardium during MI/RI (Figure 2A), which was consistent with the increase in the expression of ICAM‐1, E‐Selectin, and P‐Selectin (Figure 2D‐G). Because the adhesion molecules are involved in the chemotaxis of DCs and the process of T cell interaction, the adhesion and scrolling of DCs in endothelial cells are closely related to selectin,^26^ while DCs adhesion and cross of the vascular wall may involve the participation of intercellular adhesion molecules (ICAMs).^27^ In addition, the expression of DCs costimulatory molecules CD80 and CD86 in peripheral blood was significantly increased (Figure 2B,C), indicating that DCs was in a mature and activated state.^28^ The HMGB1 specific antibody could reduce the migration and adhesion of DCs in myocardium; inhibit ICAM‐1 obviously and reduce the expression of CD80, CD86 (*P* <0.05). The results suggest that DCs are involved in the MI/RI process and the HMGB1 signalling pathway may play an inflammatory role in DCs.

### 3.2 HMGB1 antibody attenuates myocardial injury through mediating the role of DCs regulated by the HMGB1‐TLR4 signalling pathway

**Figure 3:**
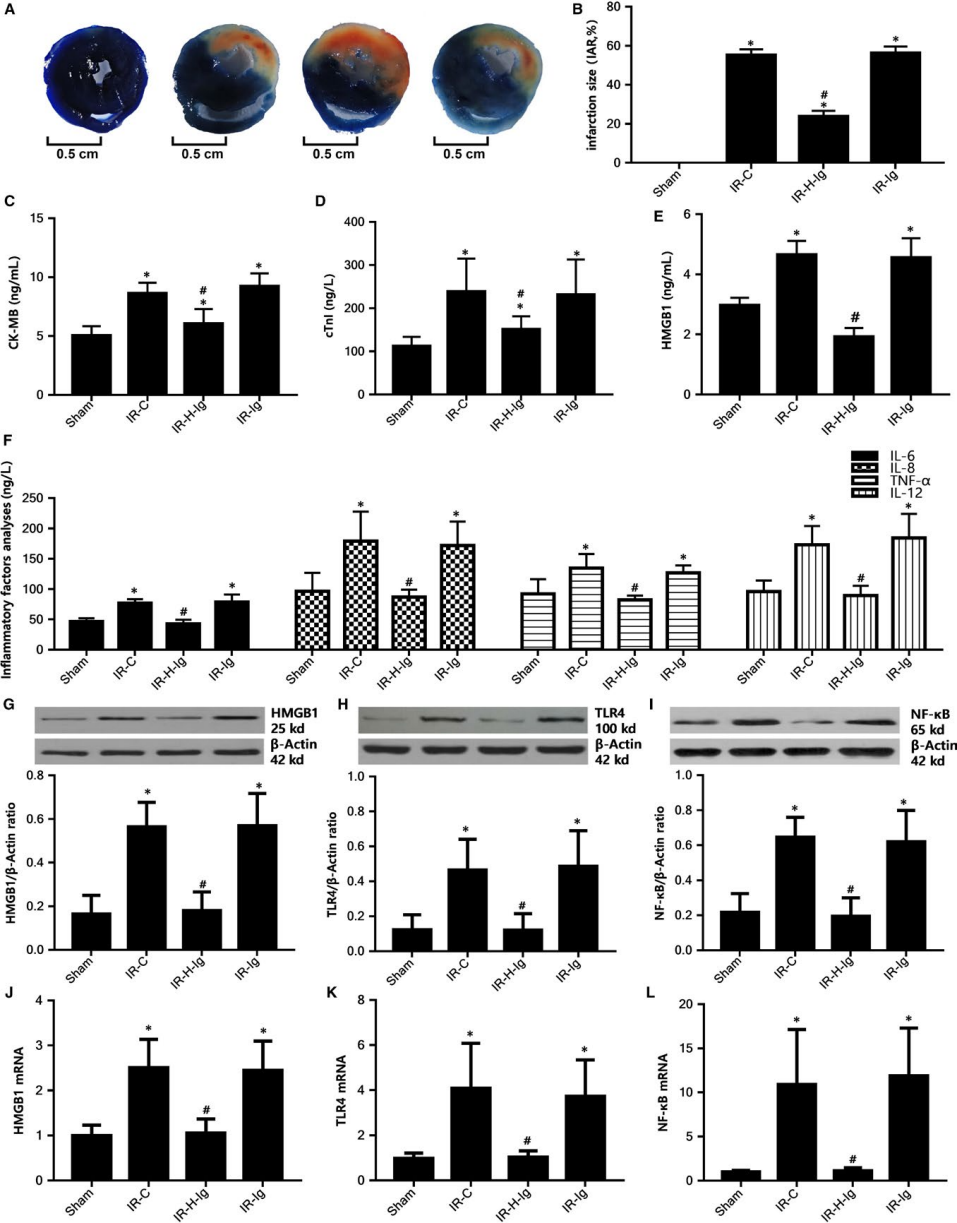
High‐mobility group protein box‐1 (HMGB1) antibody attenuates myocardial injury through mediating the role of dendritic cells (DCs) regulated by the HMGB1‐TLR4 signalling pathway. A, Comparison of myocardial infarct size among all groups (n = 6 for each group). Anti‐HMGB1 treatment reduces myocardial IR injury. Representative images of the infract area (INF: white), area at risk (AAR: red and white), and normal area (blue). B, Quantitative analysis of infarct size and the INF/AAR ratio (IAR, %). Comparison of concentrations of serum CK‐MB, cTnI, HMGB1, and IL‐6, IL‐8, TNF‐α, IL‐12 among all groups (n = 10 for each group). Blood was collected right after the IR procedure was completed. CK‐MB, cTnI, HMGB1, and IL‐6, IL‐8, TNF‐α, IL‐12 were measured as described in the Section 2. C, CK‐MB. D, CTnI. E, HMGB1. F, Inflammatory factors (IL‐6, IL‐8, TNF‐α, IL‐12). Comparison of protein and mRNA levels of HMGB1, TLR4, NF‐κB of cardiac tissues from rats of different groups (n = 10 for each group). Samples were collected right after IR procedure was completed. G, Western blot analysis of HMGB1 protein expression; H, Western blot analysis of TLR4 protein expression; I, Western blot analysis of NF‐κB protein expression; (J) relative expression of HMGB1 mRNA; (K) relative expression of TLR4 mRNA; (L) relative expression of NF‐κB mRNA. *P < 0.05, comparisons of IR‐C, IR‐H‐Ig and IR‐Ig groups with Sham group; #*P *< 0.05, comparisons of IR‐H‐Ig and IR‐Ig group with IR‐C group

**Figure 4:**
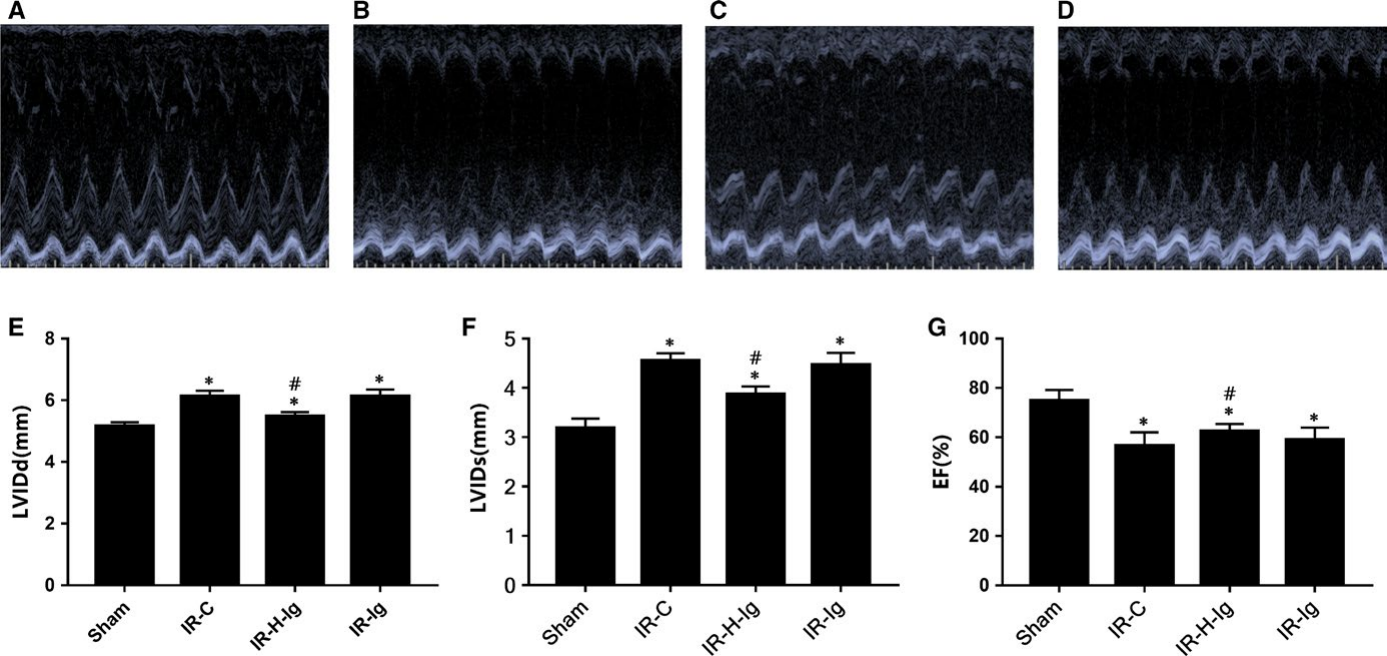
Doppler echocardiography. (A‐D) The M‐mode echocardiograms of the short‐axis midventricular view of different groups. (E‐G), The changes of left ventricular internal dimension in diastole (LVIDd), left ventricular internal dimension in systole (LVIDs) and left ventricular ejection fraction (LVEF) of different groups (n = 6 for each group). Anti‐HMGB1 treatment might improve heart function. *P < 0.05, comparisons of IR‐C, IR‐H‐Ig and IR‐Ig groups with Sham group; #*P* < 0.05, comparisons of IR‐H‐Ig and IR‐Ig group with IR‐C group

The effects of anti HMGB1 treatment on myocardial damage are illustrated in Figure 3. In our rat model, we have found that: the infarct size was grown and myocardial injury was aggravated in MI/RI. And the plasma levels of CK‐MB, cTnI, HMGB1, IL‐6, IL‐8, TNF‐α and IL‐12 were increased; The expression of HMGB1, TLR4 and NF‐κB protein and mRNA was up‐regulated in the myocardium (all *P* < 0.05). Using HMGB1 specific neutralizing antibody to antagonize HMGB1 can obviously reverse this result (all *P* < 0.05), and protect the myocardium from IRI effectively (Figure 4).

### 3.3 Regulation of HMGB1‐TLR4 signalling pathway on DCs mediates the migration, adhesion and in ischaemia‐reperfusion myocardium

**Figure 5:**
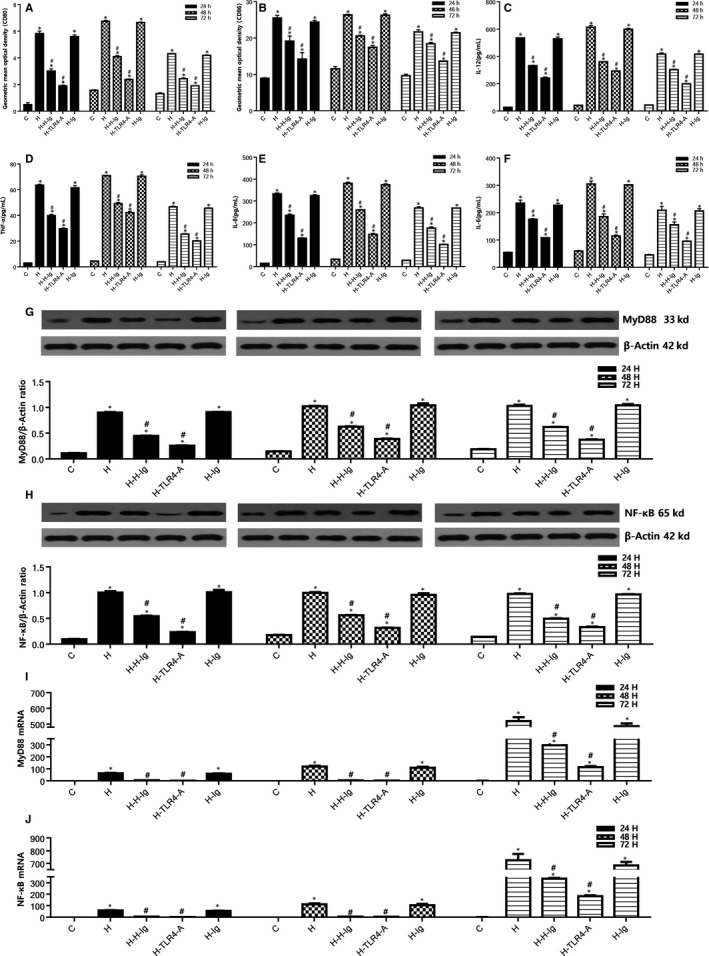
Regulation of HMGB1‐TLR4 signalling pathway on DCsHMGB1 activates DCs. Comparison of expression of CD80 and CD86 in mDCs among all groups. After isolation, culture, purification and identification of DCs, the expression of mDCs costimulatory molecules was detected by flow cytometry. A, The geometric mean optical density of CD80 in DCs; (B) the geometric mean optical density of CD86 in DCs. Comparison of 24, 48, 72 h concentrations of IL‐12, TNF‐α, IL‐8, IL‐6, in the supernatant among all groups. C, the changes of concentration of IL‐12 in the supernatant at 24, 48 and 72 h in each group; (D) the changes of concentration of TNF‐α in the supernatant at 24, 48 and 72 h in each group; (E) the changes of concentration of IL‐8 in the supernatant at 24, 48 and 72 h in each group; (F) the changes of concentration of IL‐6 in the supernatant at 24, 48 and 72 h in each group. Comparison of 24, 48, and 72 h protein and mRNA levels of MyD88, NF‐κB of mDCs from different groups. G, 24, 48, and 72 h Western blot analysis of MyD88 protein expression; (H) 24, 48, and 72 h Western blot analysis of NF‐κB protein expression; (I) 24, 48, and 72 h relative expression of MyD88 mRNA; (J) 24, 48, and 72 h relative expression of NF‐κB mRNA. *P < 0.05, comparisons of H, H‐H‐Ig, H‐TLR4‐A and H‐Ig groups with C group; #*P* < 0.05, comparisons of H‐H‐Ig, H‐TLR4‐A and H‐Ig group with H group

The activation of DCs Regulated by HMGB1‐TLR4 signalling pathway is illustrated in Figure 5. In this study, we also found that HMGB1 could increase the expression of MyD88, NF‐κB protein and mRNA, stimulate the expression of mDCs CD80, CD86, and increase the release of cytokines IL‐6, IL‐8, TNF‐α and IL‐12 by drug intervention of HMGB1, specific antibodies and TLR4 antagonists through in vitro (at 24, 48 and 72 hours after HMGB1 stimulation, all *P* < 0.05); HMGB1 specific antibody and TLR4 antagonist could significantly inhibit the expression of MyD88 and NF‐κB and the release of cytokines in the downstream of HMGB1/TLR4, and down‐regulate the expression of mDCs costimulatory molecules (at 24, 48 and 72 hours after HMGB1 stimulation, all *P* < 0.05). Previous studies have reported that the expression of mDCs surface costimulatory molecules (CD80, CD86, etc) and cytokine (IL‐12, TNF‐α, etc) on the mDCs surface can be analyzed to identify the maturation and activation of DCs.^29^ Comparison of expression of CD80 and CD86 in pbDCs (In vivo) and mDCs (In vitro) by flow cytometry see Figure S3, S4.

### 3.4 Co‐culture of DCs and CMs

The culture, purification and identification of DCs and CMs see Figure S1, S2. Flow cytometry picture of CMs apoptosis rate in co‐culture see Figure S5.

**Figure 6:**
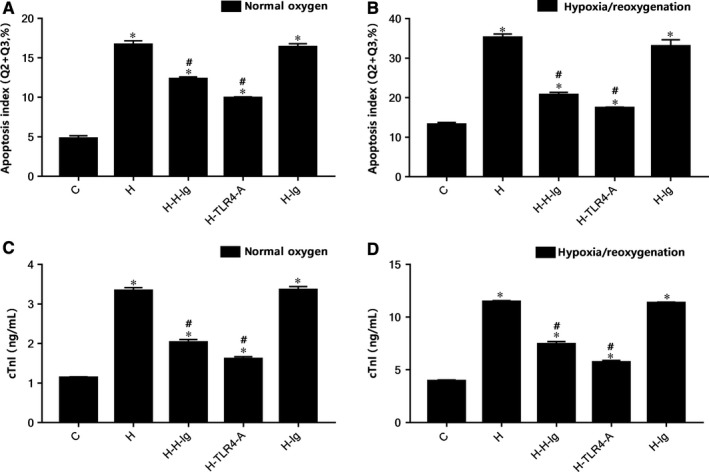
Comparison of cardiac myocyte apoptosis rate and cTnI concentration from different groups in the co‐culture of dendritic cells (DCs) and cardiac myocytes (CMs). Flow cytometry were performed. A, Apoptosis rate of CMs (Q2+Q3, %) in normal oxygen groups; (B) apoptosis rate of CMs (Q2+Q3,%) in hypoxia/reoxygenation groups; (C) CTnI concentration in normal oxygen groups; (D) CTnI concentration in hypoxia/reoxygenation groups. *P < 0.05 comparisons of H, H‐H‐Ig, H‐TLR4‐A and H‐Ig groups with C group; #*P* < 0.05, comparisons of H‐H‐Ig, H‐TLR4‐A and H‐Ig group with H group

Comparison of CMs apoptosis rate and cTnI concentration in the co‐culture of DCs and CMs are illustrated in Figure 6. In the normal oxygen and hypoxia/reoxygenation group, it was remarkable that HMGB1 stimulation produced a statistically significant decrease (*P* < 0.05) in the level of CM apoptosis rate and cTnI concentration compared with the control subgroup. Compared with the H subgroup, the apoptosis rate of CMs and cTnI in H‐H‐Ig and H‐TLR4‐Agroups were significantly decreased (*P* < 0.05).

## 4 DISCUSSION

As is known to all, inflammatory response plays an important role in IRI and it is mainly mediated by various inflammatory cells, while its potential cellular and molecular immune mechanisms are quite complex and remains unclear. Apart from macrophages, neutrophils, and lymphocytes, recently, more and more attention has been paid to the role of DCs in IRI.^30^, ^31^ Dendritic cells are the most powerful antigen presenting cells in vivo. Immature DCs are highly active to recruit macrophages, neutrophils, natural killer cells and immature lymphocytes to the inflammatory site to react in inflammatory response. Immature DCs transformed into mature DCs through different specific signal transduction mechanisms, while mature DCs no longer ingests antigen, but expresses high level MHC II and costimulatory molecules and secrets TNF‐α and IL‐12.^32^ DCs plays a unique role in combining natural and acquired immunity.

DCs are the core of early IRI innate and acquired immunity, and changes of DCs function can provide new treatment options to IRI.^33^, ^34^ There is growing evidence that DCs have a huge potential for preventing and treating of cardiovascular disease as a promising pharmacological intervention target.^35^, ^36^ However, the activation and maturation of DCs and the regulation of inflammatory mediators are influenced by many factors. One question that needs to be asked, however, is which signalling pathway mediates DCs to play a role in the process of MI/RI.

Damage‐associated molecular patterns can stimulate DCs to initiate immune response through pattern recognition receptors.^37^ Damage‐associated molecular pattern refers to cells that have changed their own conditions (such as under stress or injury) to produce recognizable danger signals, including HMGB1, defensin, etc.^38^ HMGB1 is a highly conserved DNA binding protein, which has many extracellular effects besides classical nuclear functions. There are two ways to release HMGB: one is the "active" secretion of the immune system of the body, and the other is the "passive" release of the injured, dead and apoptotic cells.^39^ The release of extracellular HMGB1 can play the role of triggering and modulating inflammation.^40^ Its uncontrolled release is likely to be damaging to the organism, which can lead to extensive inflammatory response and multiple organ dysfunction. The accumulation of HMGB1 is harmful^41^ to the organs after IRI, and blocking HMGB1 is a new strategy for the treatment of IRI.^39^ Toll like receptors, especially TLR4, are essential receptors for pattern recognition.^42^ HMGB1 can promote the inflammatory response and immune response to aggravate the organ IRI through the TLR4 signalling pathway 43, 44, 45; TLR4 is mainly expressed on DCs, macrophages and other cells, so we can hypothesize that HMGB1/TLR4 signalling pathway may regulate the function of DCs, affect the maturation, activation and chemotaxis of DCs, the expression of inflammatory factors and costimulatory molecules, and play a role in MI/RI.

In our rat model, we have found that: 1) the plasma levels of HMGB1, IL‐6, IL‐8, TNF‐α and IL‐12 were increased in the rat model of MI/RI; 2) the expression of HMGB1, TLR4 and NF‐κB protein and mRNA was up‐regulated in the myocardium; and 3) the migration, adhesion and aggregation to the myocardium CD1a^+^CD80^+^ cells increased significantly, which was consistent with the increase in the expression of ICAM‐1, E‐Selectin, and P‐Selectin. In addition, MI/RI increased the expression of DCs costimulatory molecules CD80 and CD86 in peripheral blood, increased the concentration of plasma CK‐MB and cTnI, aggravated the pathological changes of myocardium, damage cardiac function and expanded the infarct size. Using HMGB1 specific neutralizing antibody can inhibit the expression of inflammatory factors and adhesion molecules after antagonizing HMGB1, down‐regulate HMGB1, TLR4 and NF‐κB, reduce adhesion and aggregation of DCs in myocardium, and protect myocardium from injury. The results indicate that DCs are indeed involved in the MI/RI process. HMGB1 may play an inflammatory mediating role in DCs through TLR4. In addition, the expression of HMGB1 and TLR4 is positively correlated with the degree of inflammation and myocardial damage in MI/RI.

According to the experimental results, it is preliminarily concluded that HMGB1/TLR4 signalling pathway can regulate DCs maturation and activation, expression of costimulatory molecule and secretion of inflammatory mediators, to exert the regulation role of DCs on the differentiation and proliferation of T lymphocyte, thereby affecting the myocardial damage of DCs in MI/RI. The co‐culture of DCs and cardiomyocytes experiment confirmed that HMGB1/TLR4 signalling pathway mediates DCs to damage cardiomyocytes including the cardiomyocytes undergoing hypoxia/reoxygenation and increase its apoptosis. Blocking HMGB1/TLR4 signalling pathway may protect cardiomyocytes from DCs mediated cardiomyocytes hypoxia/reoxygenation injury, the co‐culture experiment provides an important theoretical basis for the role of HMGB1/TLR4 signalling pathway in the pathogenesis of DCs in the pathogenesis of MI/RI.

The inflammation is a dynamic process of change, and inflammatory cells play a variety of roles at different stages. For example, in acute renal injury, macrophages play different roles at different stages. During the initial period after reperfusion, activated macrophages within the kidney promote tubular injury, whereas at later time points, macrophages are required for tubular proliferation during normal repair.^46^ The effect of DCs is related to its maturity, activation degree and quantity.^28^ Dendritic cells not only participates in the inflammatory response after ischaemia, but also affects the healing reaction of IRI.^47^ Batal et  al^48^ reported that DCs play an important role in activating inflammatory cascade during IRI, and proposed the new idea that DCs were activated through oxidative stress. The study of Zhang et  al^49^ showed that the liver intrinsic DCs and the blood derived DCs had different functions and pointed out the importance of the local microenvironment in determining the function of DCs in the liver IRI. In our study, DCs only played an overall negative role in the early stage of MI/RI, mediated by HMGB1/TLR4 signalling pathway. Dendritic cells may play a different role as its functions or regulatory factors change over time. In addition, other leukocytes (in particular neutrophils and macrophages) that are also recruited to the ischemic myocardium based on the same mechanisms may also play a similar role. The role of DCs mediated by the HMGB1/TLR4 pathway is only a part of the overall effect, but its impact is confirmed by subsequent cell experiments.

However, the regulatory effects of HMGB1 on DCs in different concentration and different stimulation time have dual effects 29; different doses of HMGB1 have different protective effects on myocardium after MI/RI.^50^ A considerable amount of in vivo experiments also showed the complexity of the mechanism of HMGB1 functions, and some scholars believe that intravenous injection of HMGB1 before IRI has organ protection effect.^51^, ^52^ What's interesting is that Andrassy et  al^37^ found that the expression of HMGB1 was increased after MI/RI, and the damage was aggravated by the administration of HMGB1. Most of the related studies had shown that the use of HMGB1 antibodies can reduce the IRI of the organ.^53^, ^54^ However, it is also reported that the use of HMGB1 monoclonal antibodies can cause MI/RI to deteriorate.^55^ The contrary results may be related to the design conditions, the experimental conditions, the time of drug use, the dosage and the state of the disease in the model. Due to practical constraints, this study doesn't provide a research on the use of HMGB1 before and after MI/RI, and the comparison of different doses of HMGB1, which need to be further explored. Also, HMGB1 is a key downstream signal molecule of (NLRP3) inflammasome activation. An inflammasome‐independent role for NLRP3 has been demonstrated in mediating release of HMGB1 protein in activated macrophages. NLRP3 was also reported to facilitate release of pro‐inflammatory HMGB1 from activated DCs via inflammasome‐independent pathways.^56^ We will explore the specific relationship between HMGB1 and NLRP3 in MI/RI in future work to explain the complicated network if possible. In addition, this study does not explain whether the HMGB1/TLR4 signalling pathway can play a role in other cells besides the injury mediated by DCs in MIR; whether DCs is affected by other pathways in addition to the HMGB1/TLR4 pathway; whether the function of the myocardial intrinsic DCs and DCs released from peripheral blood or other organs is consistent with the function in MI/RI. All these issues need further research in the future.

In summary, in case of MI/RI, the migration, adhesion and aggregation of DCs to myocardium are increased. HMGB1/TLR4 signalling via regulating the above process, could affect the distribution of DCs in the myocardium and induce the activation and maturation of DCs, stimulate the expression of costimulatory molecules, promote the release of inflammatory factors through the MyD88/NF‐κB pathway to participate in MI/RI and aggravate myocardial injury. While antagonizing HMGB1 or blocking TLR4 could protect myocardium effectively from the MI/RI. And the ideal opportunities could be provided by Myocardial infarction and cardiovascular surgery under cardiopulmonary bypass, given the potential for ischemic heart, after reperfusion, to attenuate the damage caused by currently inevitable IRI that occurs and contributes to the increased risks of following sequelea. In conclusion, HMGB1 is a mediator of heart damage after MI/RI that operates through the TLR4 pathway to activate DCs. Administration of a neutralizing antibody to HMGB1 soon after MI/RI affords significant cardiac protection, which indicates therapeutic potential of this strategy. In this research, a new function connection was established between the HMGB1/TLR4 pathway and DCs, and it was proved that this signalling pathway can mediate the role of DCs in MI/RI. This experiment fully proves the important role and mechanism of DCs in MI/RI, which makes it possible for the follow‐up study of using DCs as the target to protect heart from MI/RI, and make it a promising future.

## 5 CONCLUSION

Myocardial ischaemia reperfusion injury is very common in clinic as the main pathophysiological process during cardiopulmonary resuscitation, thrombolysis of myocardial infarction and cardiovascular surgery under cardiopulmonary bypass. Therefore, to reduce the occurrence of MI/RI and find safer and more effective treatments has been an important research topic of the cardiovascular field.

Through in vivo and in vitro cell experiments, the following conclusions are reached in this study: In case of MI/RI, the migration, adhesion and aggregation of DCs to myocardium are increased. HMGB1/TLR4 signalling via regulating the process above, could affect the distribution of DCs in the myocardium, induce the activation and maturation of DCs, stimulate the expression of costimulatory molecules and promote the release of inflammatory factors through the MyD88/NF‐κB pathway to participate in MI/RI and aggravate myocardial injury. While antagonizing HMGB1 or blocking TLR4 could protect myocardium effectively from MI/RI. In this research, a new function connection was established between the HMGB1/TLR4 pathway and DCs, and it was proved that this signalling pathway can mediate the role of DCs in MI/RI. These findings provide ideas and theoretical basis for the prevention and treatment of MI/RI.

## ETHICS APPROVAL AND CONSENT TO PARTICIPATE

The animal procedures were approved by Wenzhou Medical University Animal Care and Use Committee (No: wydw2014‐0058), which were certified by the Chinese Association of Accreditation of Laboratory Animal Care and were consistent with the Guide for the Care and Use of Laboratory Animals (updated [2011] version of the NIH guidelines).

## AVAILABILITY OF DATA AND MATERIAL

The datasets used and/or analyzed during the current study are available from the corresponding author on reasonable request.

## CONFLICT OF INTEREST

The authors declare no conflict of interest.

## AUTHORS’ CONTRIBUTIONS

Qifeng Zhao conceived of the study, and participated in its design and coordination and helped to revised the manuscript. Jiyang Xue, Hanwei Ge and Zhiyong Lin performed the animal model experiments and collected the samples. Hanlei Wang, Wei Lin and Guowei Wu performed the solation, culture, purification and identification of rat DCs and cardiac myocyte. Jiyang Xue drafted the manuscript. Yong Liu participated in the design of the study and performed the statistical analysis. Jiyang Xue and Jie Xia prepared all figures. All authors read and approved the final manuscript.
