# Supplementary figures and images for: The role of dendritic cells regulated by HMGB1/TLR4 signalling pathway in myocardial ischaemia reperfusion injury
Source: J Cell Mol Med. 2019 Feb 19;23(4):2849–62. doi: 10.1111/jcmm.14192 (PMC6433676; doi:10.1111/jcmm.14192)

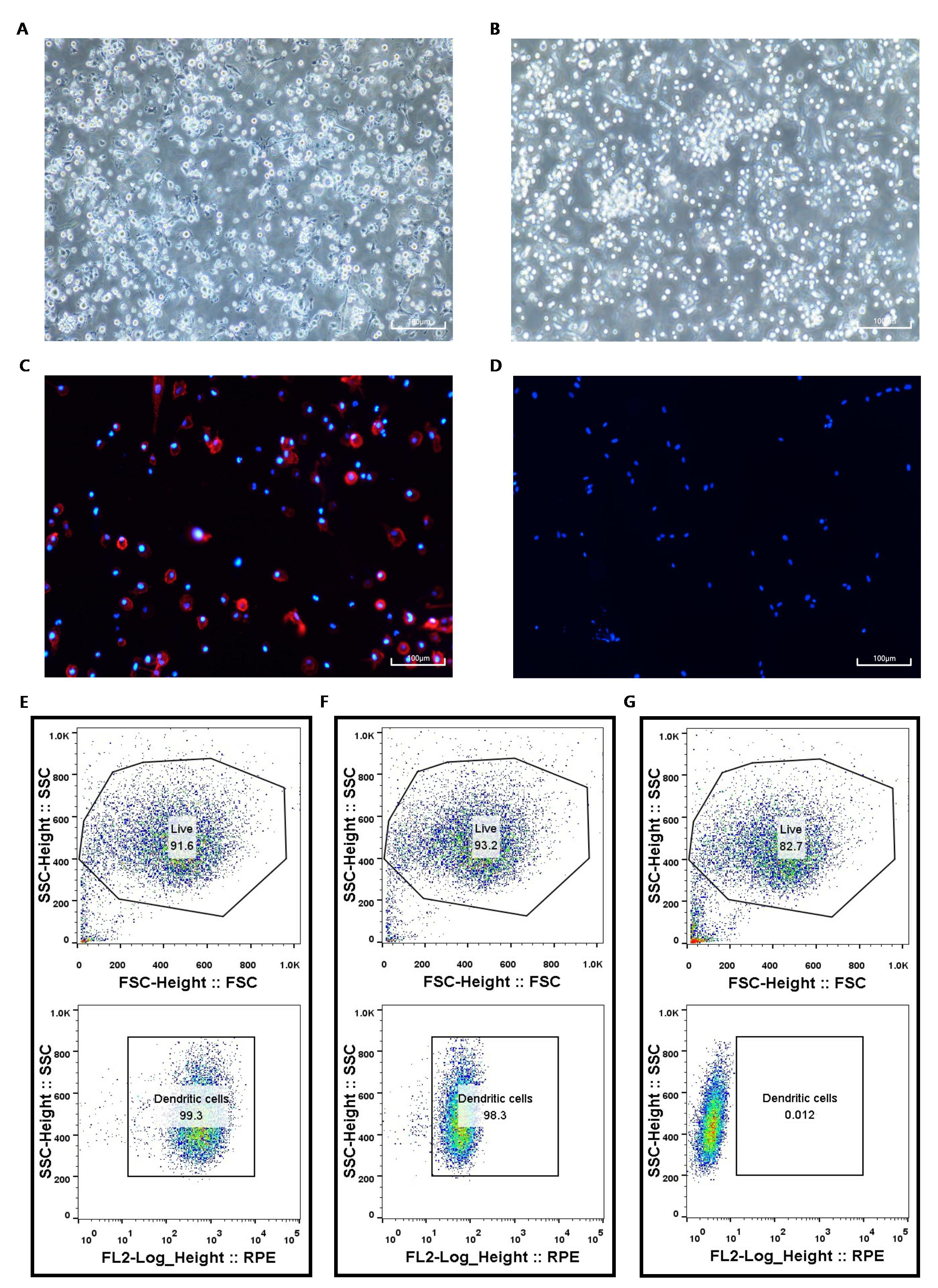

Supplement: Supplementary file 1 [file JCMM-23-2849-s001.tif]

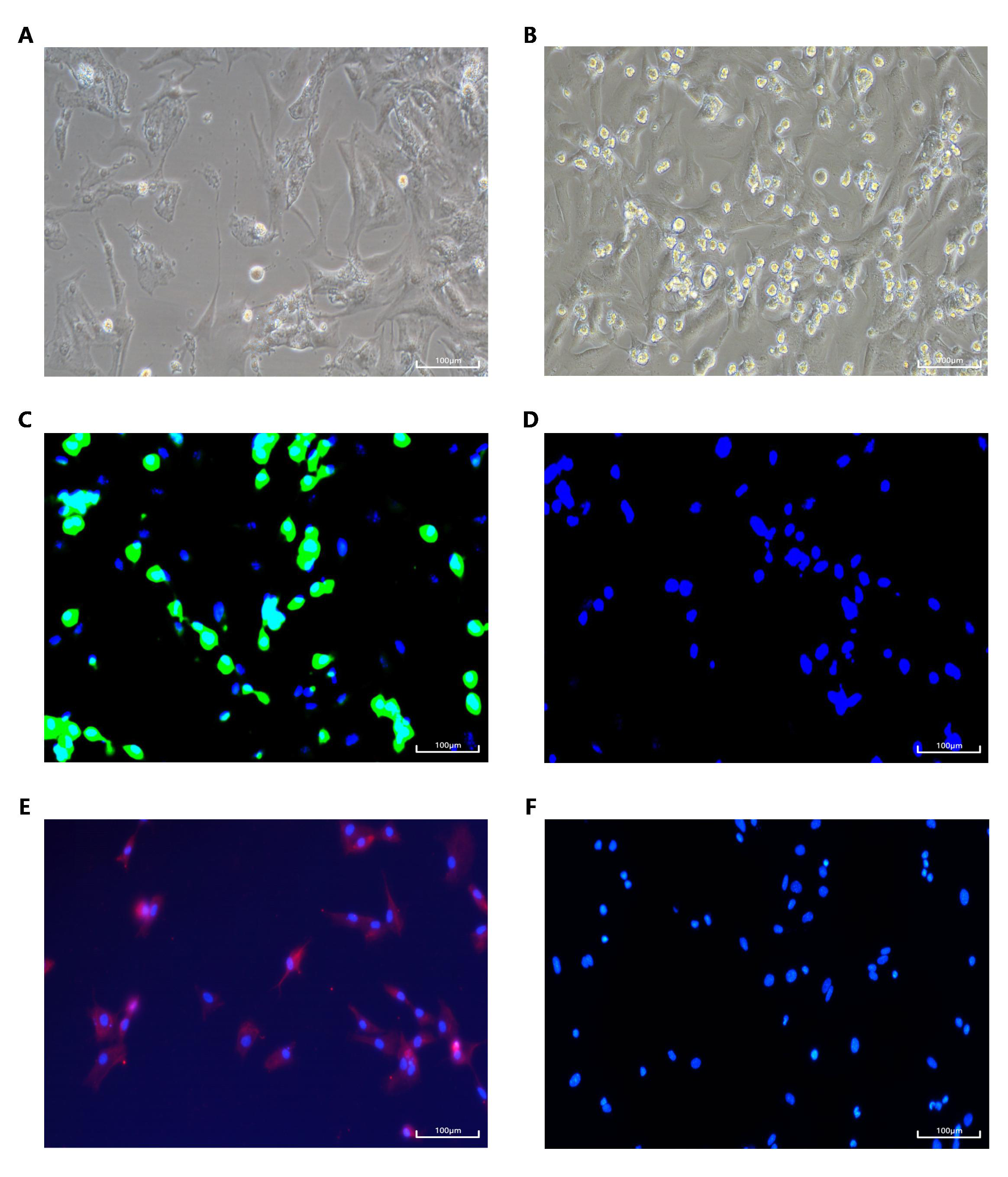

Supplement: Supplementary file 2 [file JCMM-23-2849-s002.tif]

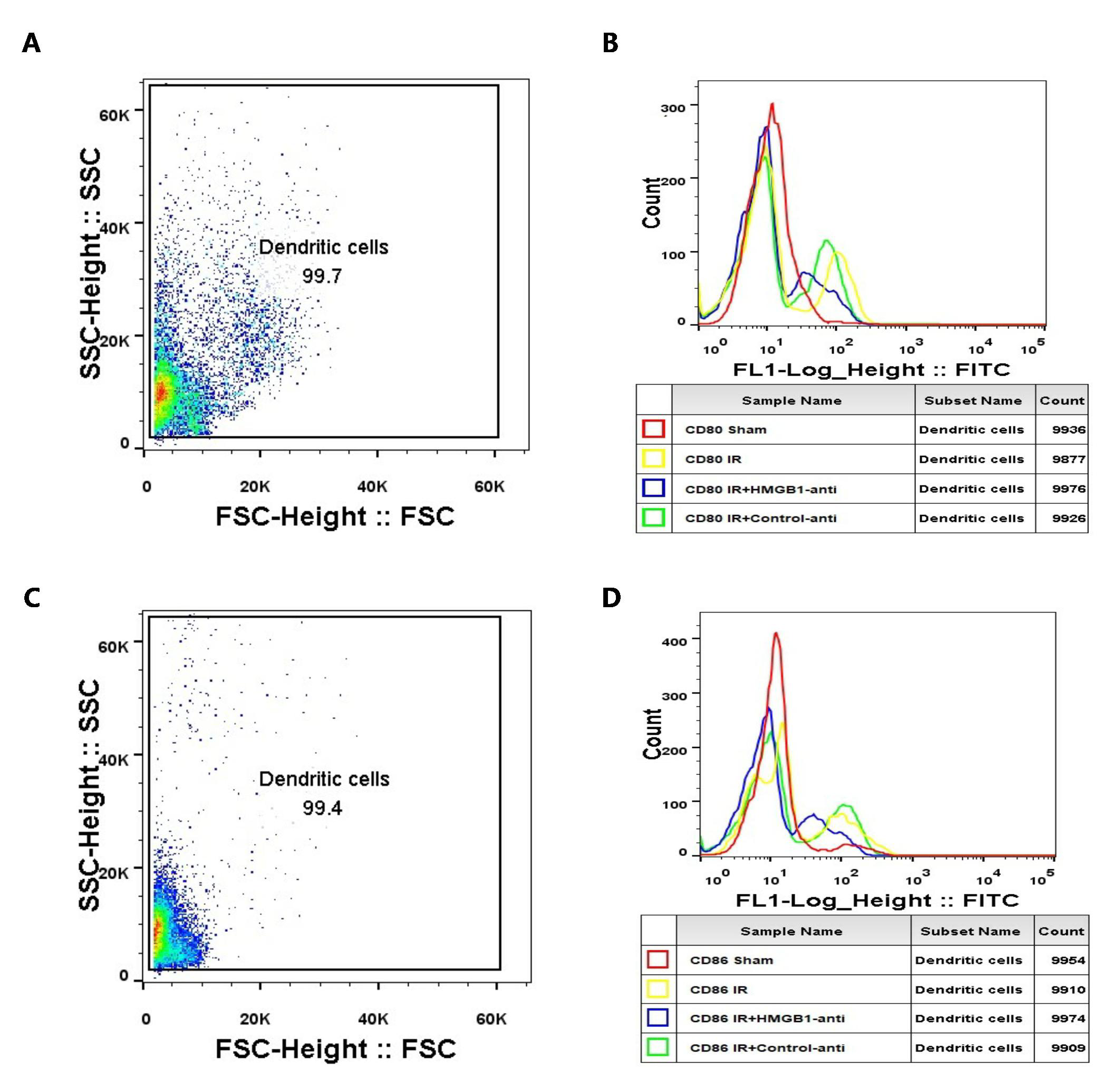

Supplement: Supplementary file 3 [file JCMM-23-2849-s003.tif]

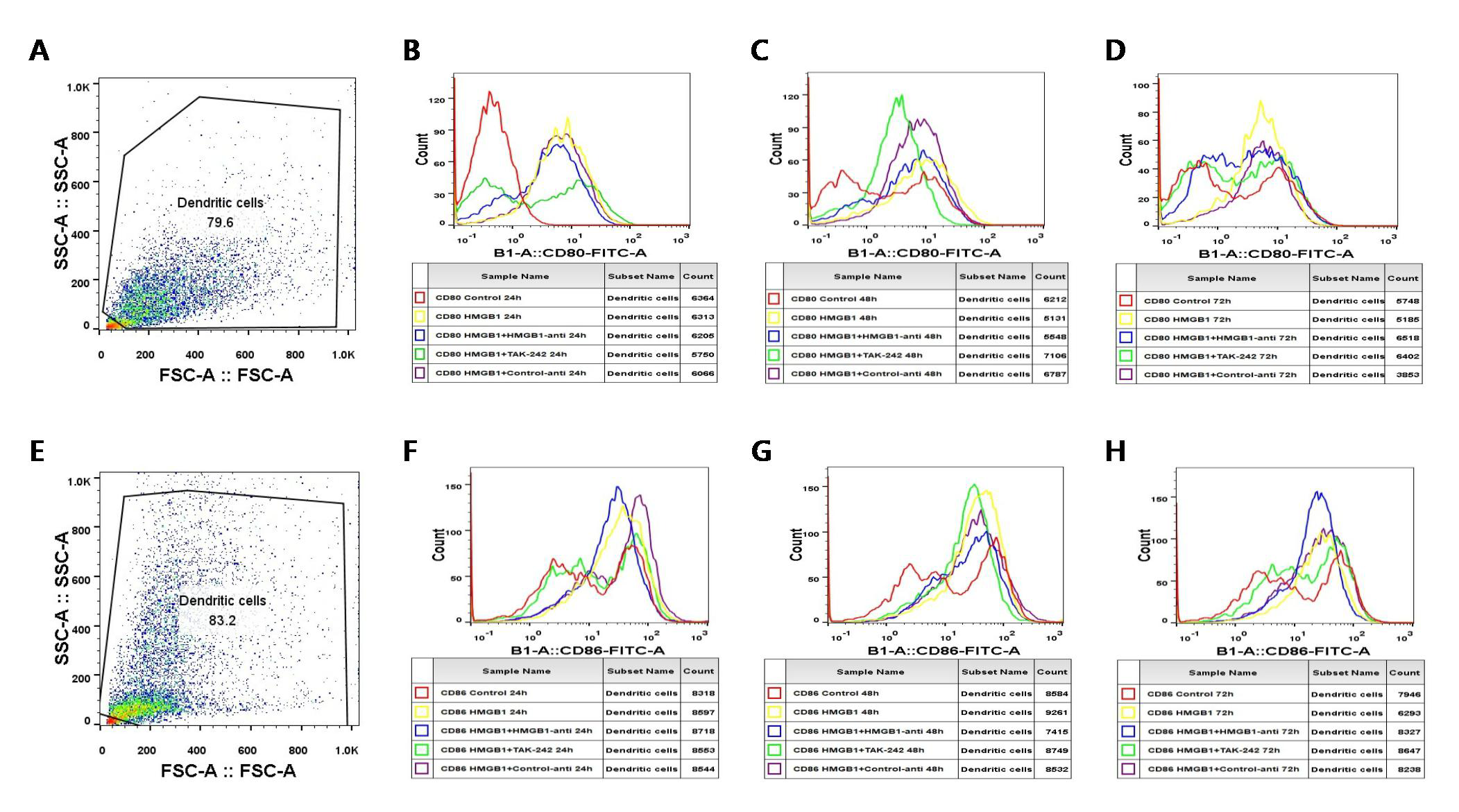

Supplement: Supplementary file 4 [file JCMM-23-2849-s004.tif]

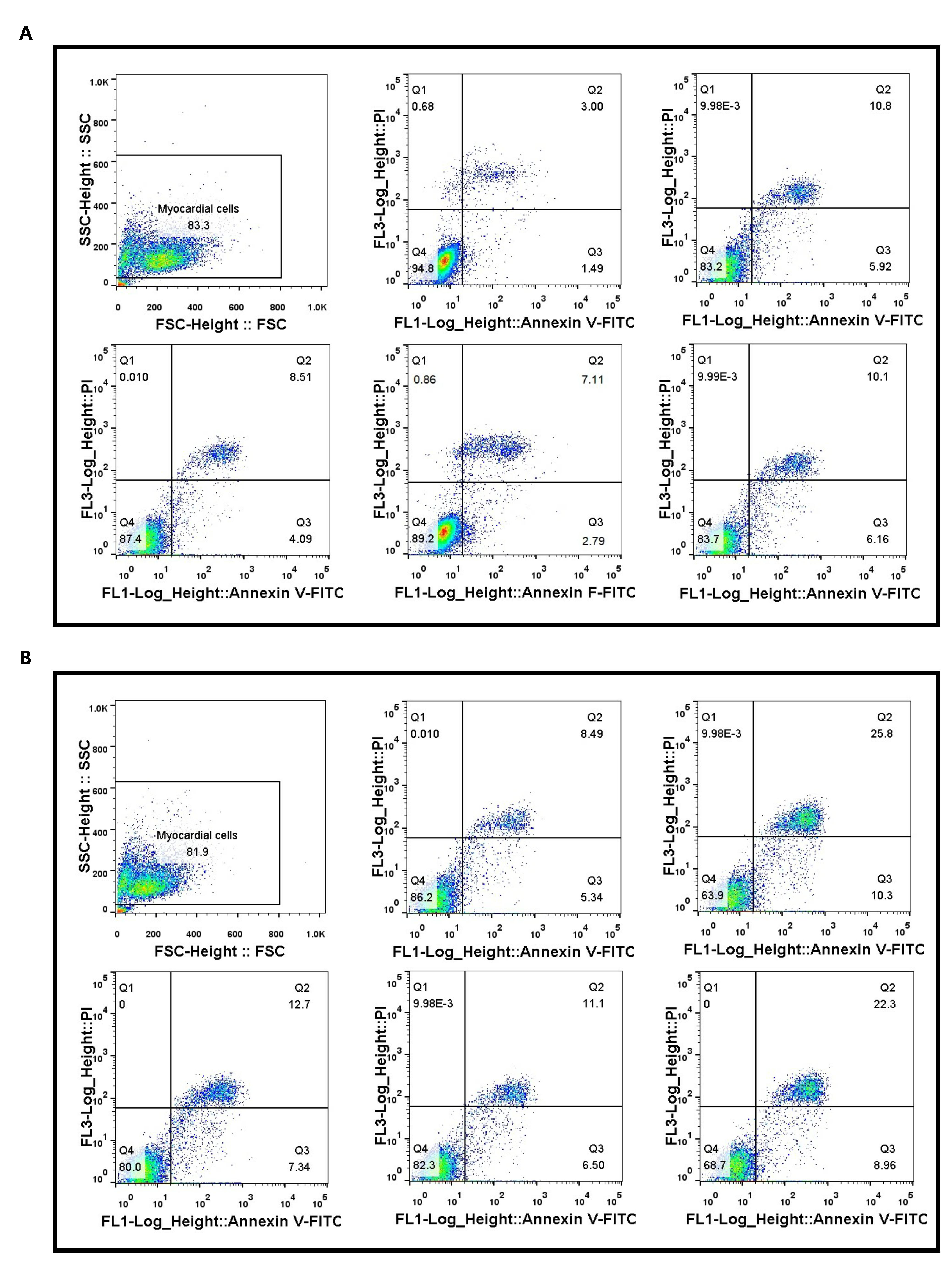

Supplement: Supplementary file 5 [file JCMM-23-2849-s005.tif]
