# Supplementary material for: The role of dendritic cells regulated by HMGB1/TLR4 signalling pathway in myocardial ischaemia reperfusion injury
Source: J Cell Mol Med. 2019 Feb 19;23(4):2849–62. doi: 10.1111/jcmm.14192 (PMC6433676; doi:10.1111/jcmm.14192)
Supplement: Supplementary file 6 [file JCMM-23-2849-s006.docx]

**Supplementary Materials**

**4. Supplementary Methods**

**4.1. Isolation, culture, purification and identification of rat dendritic cells:**

Isolation and culture of rat marrow DCs (mDCs): Adult SD rats were sacrificed and immersed in 75% alcohol for 5min, then the hind femur and tibia were removed under sterile condition. After removing the surface tissue of the femur, the ends were cut off and the contents of bone marrow were washed out with PBS solution; then the mononuclear cells were separated using the rat lymphocyte separation solution; after that, the cells were cultured in DCs complete medium containing GM-CSF and IL-4, after 6h, removed the non-adherent cells and then continued to culture, the suspended cells were collected from 3d to 9d, and the collected cells were further purified.

Purification of mDCs: The cell suspension was hung up with a certain amount of PBS, after full blending, it was sieved, counted and centrifuged, and then re-hung the cells at the bottom of the tube with the ratio of 80ul sorting buffer versus 10^7^ cells. The collected cells were incubated with the magnetic beads of rat Anti-DC (OX62, Miltenyi Biotec, Bergisch, Gladbach, Germany) and then passed through the magnetic field, using the separation principle of MACS magnetic beads (No. 130-042-302, Miltenyi Biotec, Bergisch, Gladbach, Germany) to filter cells to purify.

Immunofluorescence identification of mDCs: mDCs were detected by DCs specific protein CD11c, in which the rabbit anti rat CD11c antibody (sc-398708 PE, Santa Cruz, Dallas, TX, USA) was used as primary antibody and Alexa Fluor 594 (red fluorescence) labeled Sheep anti rabbit IgG was used as secondary antibody. DAPI at the same time was used to determine the number of cells in visual field. Red cytoplasm under the microscope represented the expression of CD11c in mDCs. In negative control group, 5% BSA instead of the primary antibody was used.

Flow identification of mDCs: After the purification was completed, the cells were treated with flow cytometry, the negative area of the fluorescence was determined by the non-antibody incubating cell upper flow cytometry; then the PE labeled CD11c antibody was incubated for 20min at 4 ℃, and the percentage of the cells that migrated into the positive region was analyzed to determine the purity of mDCs.

Isolation and culture of peripheral blood peripheral blood DCs (pbDCs): After completion of animal model test, 5~7mL of peripheral blood was collected and mononuclear cells were isolated using rat lymphocyte separation fluid. The steps of cell culture, collection, purification and identification were the same as bone marrow DCs.

**4.2. Isolation, culture, purification and identification of Cardiac myocyte (CMs) in rat:**

Isolation and culture of rat CMs: After sacrifice, the rat heart tissue and the atrium were removed, the ventricular muscle was washed and shredded and mixed with digestive juice to water bath, the upper suspension was collected, the digestive juice was added to the remaining sediment till the remain tissue was completely digested. Then, added it to the medium containing serum, collected the discontinued digestive juice to centrifugate, suspend and stain to count the cells. The cell density was adjusted to 1× 10^6^ /ml, inoculated into the uncoated culturing flask, 2ml cell suspension was added to each bottle, after standing the supernatant was collected and added to the coated culturing flask to culture.

Purification of cardiac myocytes: Primary isolated cardiomyocytes, after inoculation for 18h, removed the non-adherent cells, added 5-Brdu with a concentration of 0.1mM to the medium to inhibit the growth of fibroblasts, so that most of the fibroblasts can be killed after 48h, then after removal of 5-Brdu, continued to culture for 2 more days to restore the normal growth state of the cardiac myocytes.

Microscopical identification of cardiac myocytes: after 12h of inoculation, the morphology of cells became spindle or irregular flattened; after 24h, the spontaneous contraction of a single cell can be seen, then the cells gradually spread out and protruded the pseudo - foot to form an irregular star. On the third to fourth day, the cells interlaced into a net to form a cell monolayer or cell cluster.

Immunofluorescence identification of cardiac myocytes: using myocardial cell specific protein Troponin T and MHC antibody (Rabbit anti rat) to detect, using Sheep anti rabbit IgG labeled with Alexa Fluor 488 (green fluorescence) and Alexa Fluor 594 (red fluorescence) as secondary antibody, the green cytoplasm under microsscope represents the expression of the specific protein MHC of the cardiac myocyte was positive. At the same time, DAPI was used to determine the number of cells in the field of vision. Negative controls were incubated with 5%BSA instead of primary antibody.

**4.3. Co-culture of mDCs and CMs**

The cultured myocardial cells were purified and the mDCs cells were counted for further use. The rat cardiomyocytes were inoculated with 10 wells in the 6-well Transwell chamber with the pore size of 0.8um by the number of 200,000 cells / hole. 5 wells were incubated in the three-gas incubator with 94% N2,1% air, 5% CO2 for 3h anoxicly, and the other 5 wells were cultured under normal gas conditions for 3h (normal oxygen concentration). After the 3h of culture was completed, the upper chamber was added to each well, and the DCs 200,000 / well was inoculated in it. In the upper and lower chamber culture medium of the normal oxygen and the hypoxia / reoxygenation group, the corresponding drugs were added according to the requirements of the in vitro experimental grouping and treatment. After the drug was added, they were continuously cultured under normal gas condition for 6h, then the culture supernatant of the next chamber was taken for subsequent cTnI detection; the cells in the lower chamber were collected, and the apoptosis was detected by flow cytometry. The experiment was repeated for 3 times.

**5. Supplementary results**

**5.1. Identification of DCs**

The mDCs and peripheral pbDCs were cultured in DCs complete medium containing GM-CSF and IL-4. At the early stage, semi adherent cells were found in the bottom of the culture plate, a small number of cells were formed, cluster like, small and round, and there was no obvious protuberance in the cells; then the cell volume became larger, and there were more suspended cells in the culture medium, the cell colony scattered in it, and the morphology tended to DCs. The observed results of collected DCs on the fifth day under the phase contrast microscope are shown in Figure S1A;purified collected DCs under the phase contrast microscope are shown in Figure 1SB. After purification, the elongated fibroblast like cells in the adherent cells were decreased, and the proportion of small protruding cells (DCs) was increased in the suspension cells. The immunofluorescence showed that the cytoplasm of DCs was red (Figure S1C), which means the expression of CD11c in DCs was positive. No cytoplasmic red expression was found in the negative control with the only the blue dyed nucleus of DAPI (Figure S1D). The flow cytometry identification of mDCs showed that the purity of the purified mDCs incubated with the CD11c antibody labeled with PE was higher than 90% (Figure S1E). Similarly, the purity of the purified pbDCs was also higher than 90% (Figure S1F). The purity of DCs in negative control was very low (Figure S1G).

**5.2. Identification of CMs**

After 12h’ culture of CMs, the morphology of cells became spindle or irregular flat; after 1~2d, the cells could shrink spontaneously and gradually extend the pseudo-foot to form an irregular star (Figure S2A); after 3~4d, the cells interlaced into a network to form a cell monolayer or cell cluster (Figure S2B). 5-Bromodeoxyuridine (5-Brdu) was added to the medium to inhibit the growth of fibroblasts in order to purify CMs. The immunofluorescence showed that the cytoplasm of CMs was green (Figure S2C), which means the expression of Troponin T was positive; the cytoplasm was red (Figure S2E), which means the expression of myosin heavy chain (MHC) was positive. No green or red cytoplasm expression was found in the negative control (Figure S2D, S2F).

**5.3. Expression of pbDCs costimulatory molecules by flow cytometry (In vivo):**

The pbDCs were processed by flow cytometer sample processing if the purity was higher than 90%. The flow cytometer detection of CD80 and CD86 signal intensity (Figure S3A~S3D) was performed. These results suggest that HMGB1, as an important extracellular signal molecule, can also induce DCs maturation, promote the expression of costimulatory molecules on DCs surface, and play a regulatory role in the activation and proliferation of T lymphocytes.

**5.4. Expression of mDCs costimulatory molecules by flow cytometry (In vitro):**

The mDCs were processed by flow cytometer sample processing if the purity was higher than 90%. The flow cytometer detection of CD80 and CD86 signal intensity (Figure S4A~S4H) was performed. The expression of CD80 and CD86 was up-regulated at 24h, 48h and 72h after HMGB1 stimulation in each group, peaking at 48h, slightly decreased at 72h. The HMGB1/TLR4 signaling pathway significantly up-regulated the expression of mDCs costimulatory molecules.

**5.5. Determination of CMs apoptosis rate by flow cytometry (In Co-culture)**

After the experiment was completed, the CMs in the lower room were collected for detection of apoptosis by flow cytometry. The result of the apoptosis rate of CMs are shown in Figure S5A (normal oxygen groups) and S5B (hypoxia / reoxygenation groups). Co-culture of DCs and CMs showed that HMGB1/TLR4 signaling pathway mediates DCs damage to CMs, including CMs undergoing hypoxia / reoxygenation, and blocking HMGB1/TLR4 signaling pathway may protect CMs from injury mediated by DCs.

**6. Supplementary Figure Legends**

**Figure S1.** The culture, purification and identification of DCs. **(A)** DCs isolated and cultured for 5 days (before purification) under phase contrast microscope (200×); **(B)** Purified DCs under phase contrast microscope (200×); **(C)** Using DCs specific protein CD11c to detect, using Alexa Fluor 594 (red fluorescence) IgG as secondary antibody, using DAPI nuclear staining at the same time to determine the number of cells in visual field. Red cytoplasm under the microscope represent the expression of CD11c mDCs is positive (200×); **(D)** In negative control group, only blue DAPI nuclear staining was observed (200×); **(E)** The purity of mDCs (flow cytometer) was detected by PE labeled CD11c antibody, and the purity was higher than 90%; **(F)** PE-labeled CD11c antibody was used to detect the purity of pbDCs (flow cytometer) , purity > 90%; **(G)** In negative control group (flow cytometry), almost no DCs.

**Figure S2.** The culture, purification and identification of CMs. **(A)** Isolated and cultured CMs under microscope (200×); **(B)** CMs isolated and cultured for 4 days under microscope (200×); **(C)** Using CMs specific protein Troponin T antibody to detect, using Alexa Fluor 488 (green fluorescence) IgG as secondary antibody, using DAPI nuclear staining at the same time to determine the number of cells in visual field. Red cytoplasm under the microscope represent the expression of Troponin T of CMs is positive (200×); **(D)** In negative control, only blue DAPI nuclear staining was observed (200×); **(E)** Using CMs specific protein MHC antibody to detect, using Alexa Fluor 594 (red fluorescence) IgG as secondary antibody, using DAPI nuclear staining at the same time to determine the number of cells in visual field. Red cytoplasm under the microscope represent the MHC expression of CMs is positive (200×); **(F)** In the negative control group, only the blue DAPI nuclear staining was observed.

**Figure S3.**  Comparison of expression of pbDCs CD80、CD86 among all groups (n=10 for each group). Blood was collected right after the IR procedure was completed. After isolation, culture, purification and identification of DCs, the expression of DCs costimulatory molecules in peripheral blood of rats was detected by flow cytometry. **(A)** Delineating analysis of CDs expressing CD80 by Flow Cytometry; **(B)** Overlay histogram of the detection of CD80 expression in pbDCs by flow Cytometry; **(C)** Delineating analysis of CDs expressing CD80 by Flow Cytometry; **(D)** Overlay histogram of the detection of CD86 expression in pbDCs by flow Cytometry.

**Figure S4.** Comparison of expression of CD80 and CD86 in mDCs among all groups. After isolation, culture, purification and identification of DCs, the expression of mDCs costimulatory molecules was detected by flow cytometry. **(A)** Delineating analysis of CDs expressing CD80 by Flow Cytometry; **(B)** Overlay histogram of the detection of CD80 expression in mDCs at 24h by flow Cytometry; **(C)** Overlay histogram of the detection of CD80 expression in mDCs at 48h by flow Cytometry; **(D)** Overlay histogram of the detection of CD80 expression in mDCs at 72h by flow Cytometry; **(E)** Delineating analysis of CDs expressing CD86 by Flow Cytometry; **(F)** Overlay histogram of the detection of CD86 expression in mDCs at 24h by flow Cytometry; **(G)** Overlay histogram of the detection of CD86 expression in mDCs at 48h by flow Cytometry; **(H)** Overlay histogram of the detection of CD86 expression in mDCs at 72h by flow Cytometry.

**Figure S5.** Comparison of Cardiac myocyte apoptosis rate from different groups in the co-culture of DCs and CMs. **(A)** Flow cytometry of cardiac myocyte apoptosis in normal oxygen groups; **(B)** Flow cytometry of cardiomyocyte apoptosis in hypoxia / reoxygenation groups.
